# Ovariotomy by Enucleation; Drainage

**Published:** 1874-01

**Authors:** Geo. A. Mursick

**Affiliations:** Nyack, N. Y.


### Ovarian Tumor Removed by Enucleation; Drainage through the Douglas cul de sac, ect. Recovery.

By Geo. A. Mursick, M. D., of Nyack, N. Y.

"As the following case presents some points of practical interest, I have thought it best to place it upon record.

Mrs. G., aged about forty years, married, but never pregnant, presented herself to me about two and a half years ago for advice concerning a small tumor which she had discovered in her left iliac region. Upon examination by palpation and per vaginam, I found a tumour of the left ovary about the size of a large orange, which I advised her to let alone and await events.

The tumour slowly increased in size, so by the summer of 1872 she appeared like a woman six months pregnant; and was thought by her friends to be so. As her general health at that time was beginning to fail, I advised its removal; but she declined any operation. I did not see her again until April last, when she presented the following appearance: The tumor had largely increased in size, the abdominal walls were thin and tense, through which the outlines of several cysts could not only be felt, but could be seen. She was much amaciated, her respiration was embarrassed, and her strength was failing her. She then wanted it removed. The dangers she had to face were frankly stated to her, when she remarked that "she could die but once."

May 29th. With the assistance of Dr. John Shrady I proceeded to remove the tumor. Anæthetic, sulphuric ether. The usual incision was made through the abdominal walls below the umbilicus, and about four inches in length. Upon division of the peritoneum a large quantity of reddish-coloured serum gushed out. The largest of the cysts which presented at the opening was tapped with a large-sized trocar, and about a quart of clear amber-coloured fluid drawn off. This enabled me to introduce my hand into the abdomen, when I found the tumour largely adherent posteriorly and in the region of the pelvis. The peritoneum and the peritoneal surface of the intestines were stained a reddish-brown colour, and studded with numerous flocculi of lymph, evidence of a past peritonitis of mild character. Seven cysts were tapped successively, and their contents, a clear amber-coloured fluid, drawn off. They varied in size from an egg to a cocoanut. This reduced the size of the tumour about two-thirds. The abdominal incision was now extended upwards to two inches above the umbilicus. As an attempt to elevate the tumour from the abdominal cavity proved fruitless, I proceeded to detach it, with my fingers and the handle of a scalpel, from its adhesions and *vascular investment.* This was done without serious difficulty, and the tumour removed. *No hemorrhage occurred, and no ligatures were required.* Apprehensive lest fluid should accumulate in the abdominal cavity, after the closure of the incision, I deemed it expedient to make an opening for free drainage through the vagina. I therefore punctured the Douglas cul de sac with a large trocar, and passed one end of several strands of stout ligature silk out through the vagina, the other end out at the lower end of the abdominal incision. The two ends were knotted together externally, thus establishing a regular *seton* for drainage purposes. The abdomen was now carefully cleansed, and the incision closed with six silver sutures passed through the peritoneum, and over which was laid a cloth wet with warm water, and a binder was applied. The patient was now placed in bed; bottles of hot water were applied to her feet, and a third of a grain of morphia was given by subcutaneous injection.

The tumour, upon further examination, proved to be not only multilocular, but proliferious—that is to say, it was made up of nine primary cysts, from the interior walls of three of which numerous smaller cysts had developed, which by proliferation had nearly filled up the parent cyst, giving it the external appearance of a cystic sarcoma. The sacs and their contents weighed about thirty pounds.

29th, 10 P. M. Pulse 120; respirations 18; skin moist; mind confused from the effects of the ether; has some spasmodic twitchings of the lower extremities. Ordered ℞. Tr. opii gtt. xx; spts. frumenti ʒij, every three hours.

30th, 7 A. M. Pulse 104; skin moist; temperature normal. Drew off six ounces of urine with catheter. Says she has occasional cramps in her legs. Ordered ℞. quiniæ sulph. gr. ij; morph. sulph. gr. $\frac{1}{8}$, in solution, every three hours; also a tablespoonful of milk-punch every two hours—with cracked ice *pro re nata.* 8 P. M. Pulse 110; some fever and gastric irritability; vomited the last dose of quinia, but retained the milk-punch; has urinated twice during the day; moved the seton drainage threads backward and forwards, to keep the opening free.

31st, 7 A. M. Pulse 110 and intermittent; and complains of excessive thirst; the gastric irritability has increased; some tympanites; expression anxious; skin sallow, in fact she presents the general appearance of the initial stage of septic poisoning. Ordered ℞. Quiniæ sulph. gr. x; morphiæ sulph. gr. $\frac{1}{4}$ at once; milk-punch and ice to be continued. 12 A. M. She vomited the quinia powder, tympanites increased; complains of pain in the abdomen, and and is very restless; *slight discharge from the vagina.* Introduced a No.t's double uterine catheter through the lower end of the abdominal incision, and washed out the abdomen with the following solution: ℞. Sodæ chlorid. gr. xx; acid. carbolic. gr. v; aquæ bul. Oj. The return discharge was of a dirty red colour. 7 P. M. Pulse 110, and feeble; the stomach has rejected everything swallowed, and she is much prostrated. the tongue is becoming red and dry. Ordered ℞. Quiniæ sulph. gr. iv: morphiæ sulph. gr. ⅛, by subcutaneous injection; and sodæ hyposulphit. gr. xx, every three hours in water; also an enema of beef-tea, containing spirits. frumenti ℥j.

June 1, 7 A. M. Pulse 110, and of good volume; expression less anxious. The gastrice irritability is lessened; but she vomits the soda hyposulphite; there is a very free discharge of red serum per vaginam. Syringed out the abdomen as before; also the vagina with a weak and warm solution of acid. carbolic. Ordered ℞. Quiniæ sulph. gr. iv; morphiæ sulph. gr. ⅛, every six hours, by subcutaneous injection; also an anema of beef-tea, containing spts. vini. gal. ℥ss, three times daily, and as much cracked ice, per orem, as she chooses to take. 7 P. M. Fever less; some retching, and she spits up a great deal of viscid mucus; tympanites increased. Syringed out the abdomen and vagina as before, and continued the hypodermic injection of quinia and morphia.

2d, 3d, and 4th. The gastric irritability has been excessive, and the constant retching and ejection of viscid mucus tinged with bile have induced great prostration; otherwise she has remained pretty much in the same condition, and the same treatment has been continued. The discharge per vaginam has been quite free, and the seton threads have been drawn backwards and forwards twice daily to keep the opening free.

5th. 8 A. M. Her general condition has much improved; pulse 98; no fever; tongue red, but more moist; the gastric irritability has nearly ceased, and she expresses a de ire for food; the discharge per vaginum is much less, and the tympanites is slight; syringed out the vagina, etc., ℞. Tinct. ferri chlor. gtt. xx.; potass. chlorat. gr. x, every six hours. To have chicken-broth and milk-punch. The abdominal incision has entirely healed except at its lower end; removed all the sutures. 7 P. M. Some fever; pulse 104; discharge per vaginam free; there is a small bed-sore forming over the sacrum.

6th. 8 A. M. Pulse 86; she is steadily improving and is able to take a moderate quantity of fluid food and milk-punch, which the stomach retains together with the medicine. ℞ Quiniæ sulph.. gr. v, in one dose daily in addition to the iron, etc. 7 P. M. The discharge from the vagina is slight, and the abdomen is quite flaccid; is doing well.

7th. 8 A. M. Pulse 94; complains of a great deal of flatus; her bowels have not been moved since the operation; removed the seton threads. ℞. Ol. ricini ℥j; other treatment continued.

7 P. M. Bowels have moved three times; pulse 90; is sweating profusely.

8th. Pulse 86; is doing well, but is excessively weak; the discharge per vaginam is very slight. ℞. Acid. phosphoric. dilut. gtt. xx; strychniæ gr. $\frac{1}{20}$, every six hours, and brandy four ounces daily made into milk-punch, and as much beef-tea as she will take.

11th. She has steadily continued to improve, though her bowels have been rather free for the past twenty-four hours; the stools are black and tarry; the acid. phosphor. and strychnia were suspended, and ferri et potass. tart. gr. v, and quiniæ sulph. gr. ij, three times daily, substituted; the diarrhœa is controlled by tinct. opii deodorat. gtt. xx pro re nata.

28th. She continues to improve, but regains her strength and appetite slowly; some slight discharges have occurred per vaginam, but they have now ceased; she sits up part of the time, and occasionally walks across the room. ℞. Quiniæ sulph. gr. ij; strychniæ gr. $\frac{1}{20}$; three times daily in solution.

July 14th. Has continued to do well, and is now up and about the house attending to her household duties; her general health is steadily improving, and she states that she feels much better than she has done for the past three years.

Aug. 1st. Health perfect; is growing quite fleshy; menstruates regularly, and has hopes of future offspring.

I have reported this case in detail because of its points of practical interest, viz:—

1. The removal of the tumor by enucleation.
2. The establishment of free drainage per vagina by the seton.
3. The administration of Medicine by subcutaneous injection, in consequence of the excessive gastric irritability.
4. The support of the patient by nutritive enemata.
5. The value of quinia in the prevention and cure of septicemia.

Since Dr. Miner reported his case a number of ovarian tumours have been removed by enucleation, and I have no doubt but that further experience will prove this method of procedure to be one of great value, especially in cases where the peduncle is short, or, as in the above case, where there was practically no peduncle. It dispenses with the use of clamps, ligatures, *et id omne genus*. The ruptured vessels of the investment of the tumour bleed but little, if any, and a slight oozing of blood is of much less consequence, where free drainage is established, than the application of ligatures of any kind, which *are not always* sacculated, and which often induce the formation of abscesses and other obnoxious sequelæ.

Dr. Sims has recently called attention to the value of free drainage per vaginam as a preventive of septicemia after ovarian operations, and with his usual ingenuity has devised several instruments to facilitate it, as he was not satisfied with Chassaignac's drainage tubes. The procedure adopted by myself was a very simple one, and in this case proved effective, not only in affording free drainage per vaginam, but in keeping open the lower end of the abdominal incision, and thus avoiding the use of tents, which are occasionally required for that purpose. By the aid of Nott's double uterine catheter introduced through the opening, the abdomen can at any time be thoroughly syringed out with an antiseptic or other wash, as has been often done by Dr. Peaslee, to whom we are indebted for this procedure.

The value of quinia as a preventive and in the treatment of septicemia has been fully established, and I need not dwell upon that point, but I would especially call attention to its administration by subcutaneous injection, in cases accompanied by excessive gastric irritability. For several days my patient's life "hung by a thread," the stomach rejecting everything; but by this method it was administered with facility and its action was prompt. To it and the administration of sufficient nutriment by the rectum during the days of gastric irritability, I believe my patient owes her life more than to anything else.

I have given quinia by the rectum both in septicemia and in pyœmia during my military service, but its absorption into the system was so slow that but little if any good was accomplished by it. Had I given it at those times by subcutaneous injection, I have no doubt that my success would have been greater."—*American Journal Med. Sciences.*

